# Supplementary material for: A Critical View of the Application of the APEX Software (Aqueous Photochemistry of Environmentally-Occurring Xenobiotics) to Predict Photoreaction Kinetics in Surface Freshwaters
Source: Molecules. 2019 Dec 18;25(1):9. doi: 10.3390/molecules25010009 (PMC7017383; doi:10.3390/molecules25010009)
Supplement: Supplementary file 1 [file molecules-25-00009-s001.zip › 3.2.4_gcc-4.4.0.zip!/3.2.4_gcc-4.4.0/3.2.4_gcc-4.4.0/doc/octave/html/interpreter/Documentation-and-Test-of-Oct_002dFiles.html]

Documentation and Test of Oct-Files - Untitled


Previous: Exception and Error Handling in Oct-Files,
Up: Oct-Files


---

#### A.1.13 Documentation and Test of Oct-Files

The documentation of an oct-file is the fourth string parameter of the
`DEFUN_DLD` macro. This string can be formatted in the same manner
as the help strings for user functions (Documentation Tips),
however there are some issue that are particular to the formatting of
help strings within oct-files.

The major issue is that the help string will typically be longer than a
single line of text, and so the formatting of long help strings need to
be taken into account. There are several manners in which to treat this
issue, but the most common is illustrated in the following example

```
     DEFUN_DLD (do_what_i_want, args, nargout,
       "-*- texinfo -*-\n\
     @deftypefn {Function File} {} do_what_i_say (@var{n})\n\
     A function that does what the user actually wants rather\n\
     than what they requested.\n\
     @end deftypefn")
     {
     ...
     }
```

where, as can be seen, end line of text within the help string is
terminated by `\n\` which is an embedded new-line in the string
together with a C++ string continuation character. Note that the final
`\` must be the last character on the line.

Octave also includes the ability to embed the test and demonstration
code for a function within the code itself (Test and Demo Functions).
This can be used from within oct-files (or in fact any file) with
certain provisos. Firstly, the test and demo functions of Octave look
for a `%!` as the first characters on a new-line to identify test
and demonstration code. This is equally a requirement for
oct-files. Furthermore the test and demonstration code must be included
in a comment block of the compiled code to avoid it being interpreted by
the compiler. Finally, the Octave test and demonstration code must have
access to the source code of the oct-file and not just the compiled code
as the tests are stripped from the compiled code. An example in an
oct-file might be

```
     /*
     
     %!error (sin())
     %!error (sin(1,1))
     %!assert (sin([1,2]),[sin(1),sin(2)])
     
     */
```
